# ﻿On the taxonomy of the subgenera *Tatsipolia*, *Chalapolia*, and *Kitapolia* of the genus *Dasypolia* Guenée with the description of six new species from southern Xizang, China (Insecta, Lepidoptera, Noctuidae)

**DOI:** 10.3897/zookeys.1244.152267

**Published:** 2025-07-11

**Authors:** Enyong Chen, Zhaohui Pan, Anton V. Volynkin, Aidas Saldaitis, Balázs Benedek, Yonghong Zhou

**Affiliations:** 1 Key Laboratory of Biodiversity and Environment on the Qinghai-Xizang Plateau, Ministry of Education, School of Ecology and Environment, Xizang University, Lhasa, 850000, China; 2 Tibetan Yani Wetland Ecosystem National Observation and Research Station, Xizang University, Lhasa, 850000, China; 3 Key Laboratory of Forest Ecology in Tibet Plateau, Institute of Plateau Ecology, Tibet Agricultural and Animal Husbandry University, Ministry of Education, Linzhi 860000, China; 4 Altai State University, Lenina Avenue, 61, RF-656049, Barnaul, Russia; 5 Nature Research Centre, Akademijos str., 2, LT-08412, Vilnius-21, Lithuania; 6 Szőlőhegy, 8497/7, H-7700, Mohács, Hungary

**Keywords:** Antitypina, new synonym, Noctuinae, owlet moth, Xylenini

## Abstract

Species of the subgenus Tatsipolia Benedek, Behounek, Floriani & Saldaitis, 2011 of the genus *Dasypolia* Guenée, 1852 are reviewed and the genus-group names *Chalapolia* Benedek, Behounek, Floriani & Saldaitis, 2011 and *Kitapolia* Benedek, Behounek, Floriani & Saldaitis, 2011 previously considered as distinct subgenera of *Dasypolia* are synonymised with *Tatsipolia*. Six new species are described from southern Xizang, China: Dasypolia (Tatsipolia) polymorpha**sp. nov.**, Dasypolia (Tatsipolia) intermedia**sp. nov.**, Dasypolia (Tatsipolia) amoena**sp. nov.**, Dasypolia (Tatsipolia) diffusa**sp. nov.**, Dasypolia (Tatsipolia) luxuriosa**sp. nov.**, and Dasypolia (Tatsipolia) ultramontana**sp. nov.** Adults and male and female genitalia of all species in Dasypolia (Tatsipolia) are illustrated.

## ﻿Introduction

*Dasypolia* Guenée, 1852 is a large owlet moth genus distributed in Palearctic Region and reaching its greatest diversity in high mountain areas of Asia. The genus belongs to the subtribe Antitypina of the tribe Xylenini of the subfamily Noctuinae (Lafontaine and Schmidt 2010; Zahiri et al. 2011; Keegan et al. 2021). Many species of *Dasypolia* were described during the last two decades (Nupponen and Fibiger 2006; Ronkay et al. 2010, 2014, 2023; Benedek et al. 2011, 2014; Volynkin 2012; Benedek and Saldaitis 2014; Chen et al. 2022; Pekarsky and Pekarska 2022). However, the fauna and taxonomy of the genus sensu lato are understudied in the highest mountain regions of eastern Himalaya and southern part of the Tibetan Plateau and it is likely that additional undescribed species will be found.

The genus is currently subdivided into nine subgenera (Benedek et al. 2011, 2016; Benedek and Saldaitis 2014; Ronkay et al. 2014; Gordeev et al. 2023; Ronkay and Ronkay 2023), some of which are substantially different from the nominate one in their genitalia morphology. The subgenus Tatsipolia Benedek, Behounek, Floriani & Saldaitis, 2011 was erected to solely include Dasypolia (Tatsipolia) ruficilia Benedek, Behounek, Floriani & Saldaitis, 2011 (Benedek et al. 2011). Subsequently, another species of the genus, *Dasypoliavignai* L. Ronkay & Zilli, 1993 was assigned to *Tatsipolia* after the discovery of the male of the species (Benedek and Saldaitis 2014), and eight years later, the subgenus was reviewed by Chen et al. (2022), who described two additional species from southern Xizang Province of China. Two other subgenera morphologically similar to *Tatsipolia*, *Chalapolia* Benedek, Behounek, Floriani & Saldaitis, 2011 and *Kitapolia* Benedek, Behounek, Floriani & Saldaitis, 2011 were erected to solely include Dasypolia (Chalapolia) brandstetteri Benedek, Behounek, Floriani & Saldaitis, 2011 and Dasypolia (Kitapolia) kita Benedek, Behounek, Floriani & Saldaitis, 2011, respectively (Benedek et al. 2011), and both remained monotypic up to date.

During further entomological studies in the southern Xizang, extensive additional materials on *Dasypolia* sensu lato comprising six unidentified species were collected by the senior author of the present paper. After comparing the male genitalia structures of those species with other species in the genus, they proved to belong to the subgenera *Tatsipolia* and *Chalapolia* and express significant distinctive characters from known taxa and therefore represent species new to science. Additionally, the detailed examination of their morphology led to the conclusion that the type species of *Tatsipolia*, *Chalapolia* and *Kitapolia* display no fundamental differences in their genitalia of both sexes and are nothing more than species groups within the same subgenus. In the present paper, *Tatsipolia* is selected as the senior genus-group name for this complex with *Chalapolia* and *Kitapolia* being synonymised with it, all species included into the subgenus Tatsipolia are reviewed and six new species are described.

## ﻿Materials and methods

Abbreviations of the depositories used:

**AFM** research collection of Alessandro Floriani (Milan, Italy);

**ASV** research collection of Aidas Saldaitis (Vilnius, Lithuania);

**BBT** research collection of Balázs Benedek (Mohács, Hungary);

**HNHM**Hungarian Natural History Museum (Budapest, Hungary);

**MH/HNHM** collection of Márton Hreblay in Hungarian Natural History Museum (Budapest, Hungary);

**TU** Tibet University (Lhasa, China);

**TAAHU** Tibet Agricultural and Animal Husbandry University (Linzhi, China);

**ZSM**Bavarian State Collection of Zoology (Zoologische Staatssammlung München, Munich, Germany).

Other abbreviations used: **GB** = genitalia slide prepared by Gottfried Behounek; **HT** = holotype; **JB** = genitalia slide prepared by János Babics; **PT** = paratype; **RL** = genitalia slide prepared by László Ronkay.

The genitalia of all specimens deposited in TU and TAAHU were dissected by the senior author of the present paper applying standard methods of preparation (Lafontaine et al. 1987; Kononenko 2010) and preserved in glycerol in micro vials pinned under the specimens. The photos of adults were taken using the Canon 5DIV camera while the photos of genitalia were taken using the LEICA EZ4 HD camera. All pictures were processed using the Adobe PHOTOSHOP CC 2018 software.

For the holotype label citations, information provided in quotation marks is transcribed verbatim. Different labels are separated by a slash (“/”) while the different lines of the same label are separated by a vertical bar (“|”). Any additional data are provided in square brackets. The content of the paratype labels is edited and unified. The male and female genitalia terminology follows Volynkin (2024) and Kononenko (2010).

## ﻿Results

### ﻿Family Noctuidae Latreille, 1809

**Subfamily Noctuinae Latreille, 1809**

**Tribe Xylenini Guenée, 1837**

**Subtribe Antitypina Forbes & Franclemont, 1954**

**Genus *Dasypolia* Guenée, 1852**

#### ﻿ Subgenus Tatsipolia

Taxon classificationAnimaliaLepidopteraNoctuidae

Benedek, Behounek, Floriani & Saldaitis, 2011

FEE9F1FD-9A60-5A71-BC20-997C8FE257FC

Dasypolia subgenus Tatsipolia , Benedek et al., 2011: 108 (type species: Dasypolia (Tatsipolia) ruficilia Benedek, Behounek, Floriani & Saldaitis, 2011, by original designation).
= DasypoliasubgenusChalapolia, Benedek et al., 2011: 109 (type species: Dasypolia (Chalapolia) brandstetteri Benedek, Behounek, Floriani & Saldaitis, 2011, by original designation), syn. nov.
= DasypoliasubgenusKitapolia, Benedek et al., 2011: 110 (type species: Dasypolia (Kitapolia) kita Benedek, Behounek, Floriani & Saldaitis, 2011, by original designation), syn. nov.

##### Diagnosis.

Species of D. (Tatsipolia) are relatively small owlet moths externally reminiscent of members of the genus *Cteipolia* Staudinger, 1896 (see Gordeeva et al. 2023; Volynkin et al. 2024), from which D. (Tatsipolia) differs clearly in the genitalia morphology of both sexes. The male genital capsule ground plan of D. (Tatsipolia) is similar to *Dasypolia* s. str. (e.g., see Ronkay et al. 2001, 2014) and the main differences are found in the phallus and vesica: in D. (Tatsipolia), the phallus carina is smooth and the vesica bears one or two clusters of spine-like cornuti medially whereas the carina of *Dasypolia* s. str. bears a dentate plate and the vesica is unarmed. In the female genitalia, D. (Tatsipolia) has asymmetrical anterior sclerotisations of the ductus bursae (it is evenly sclerotised in the similar genus) and a reduced appendix bursae, which is well-developed and semiglobular or conical in *Dasypolia* s. str.

##### Distribution.

Species of the genus are known only from south-western China (Sichuan and southeastern Xizang).

### ﻿Taxonomic content of Dasypolia (Tatsipolia)

The D. (T.) vignai species group:

- D. (T.) polyformis sp. nov.
- D. (T.) cerritula Chen, Pan, Volynkin, Saldaitis & Benedek, 2022
- D. (T.) sejilaensis Chen, Pan, Volynkin, Saldaitis & Benedek, 2022
- D. (T.) vignai L. Ronkay & Zilli, 1993
- D. (T.) ruficilia Benedek, Behounek, Floriani & Saldaitis, 2011
- D. (T.) intermedia sp. nov.

The D. (T.) nivalis species group:

- D. (T.) brandstetteri Benedek, Behounek, Floriani & Saldaitis, 2011
- D. (T.) amoena sp. nov.
- D. (T.) diffusa sp. nov.
- D. (T.) luxuriosa sp. nov.
- D. (T.) ultramontana sp. nov.
- D. (T.) nivalis Hreblay & L. Ronkay, 1995

The D. (T.) kita species group:

- D. (T.) kita Benedek, Behounek, Floriani & Saldaitis, 2011

### ﻿Taxonomic accounts

#### ﻿The D. (T.) vignai species group

**Diagnosis.** The male genitalia of the species group are characterised by the reduced harpe, which may be present as a short tubercle. In the female genitalia, the ostium bursae is broad, the ductus bursae has three regions of sclerotisation (the posterior of which forms a ventral margin of the ostium bursae), and the anterior (dilated) section of the corpus bursae is short (equal to the ovipositor length or shorter).

##### ﻿ Dasypolia (Tatsipolia) polyformis sp. nov.

Taxon classificationAnimaliaLepidopteraNoctuidae

72773D18-EA24-56B1-9028-C8C06785295D

https://zoobank.org/42629A7E-D40F-4E8B-8692-C6F7D7A758B0

Figs 1–9 , 39–41 , 59 , 60

###### Type material.

**Figures 1–10.**
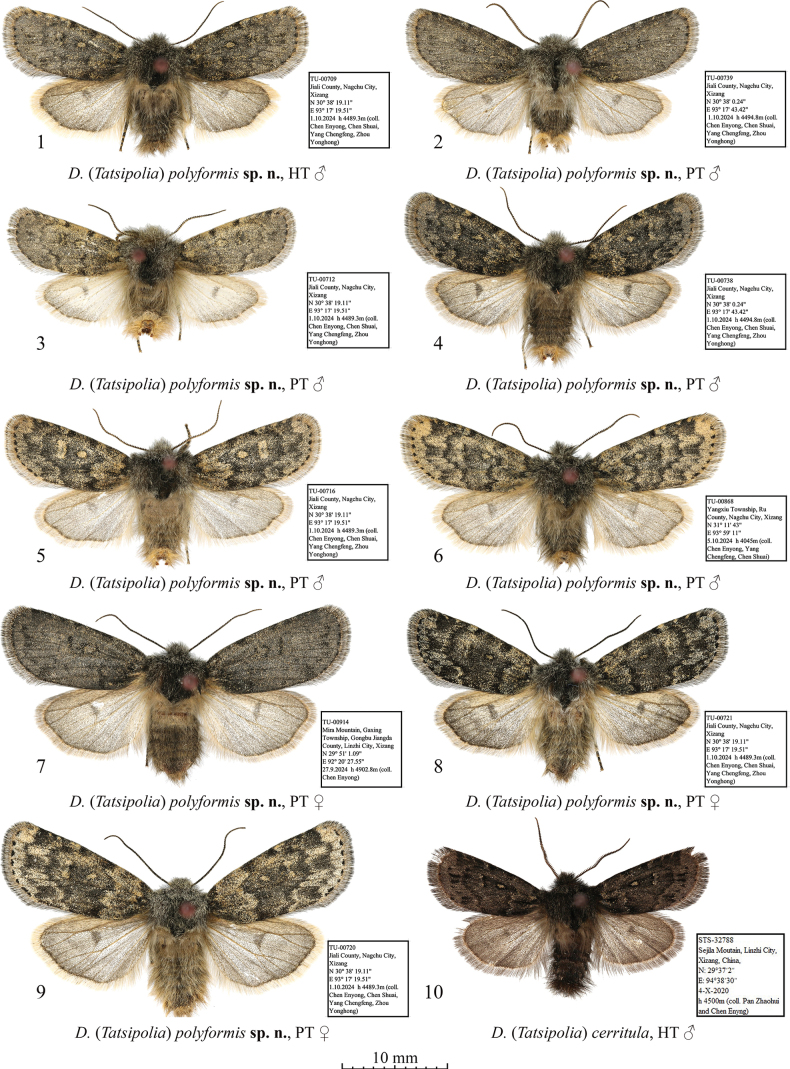
Adults of Dasypolia (Tatsipolia) spp. Depositories of the specimens: **1–9.** In TU; **10.** In TAAHU.

***Holotype*** (Figs 1, 39): China • ♂, “TU-00709 | Jiali County, Nangchu City, | Xizang | N 30° 8' 19.11" | E 93°17'19.51" | 1.10[x].2024 h [altitude] 4489.3 m (coll. [leg.:] | Chen Enyong, Chen Shuai, | Yang Chengfeng, Zhou | Yonghong)” (TU). ***Paratypes*** (15 ♂♂, 9 ♀♀, all in TU). China • 2 ♂♂, 5 ♀♀, same data as in holotype, unique IDs: TU-00711, 00712, 00714 to 00716, 00720, 00721; • 3 ♂♂, 1 ♀, same data as previous but 30°38'0.24"N, 93°17'43.42"E, 4494.8 m, unique IDs: TU-00730, 00738 to 00740; • 1♂, 1♀, same data as previous but 30°37'51.35"N, 93°19'8.07"E, 4427 m, unique IDs: TU-00744, 00747; • 3 ♂♂, 1 ♀, Yangxiu Township, Ru County, Nangchu City, Xizang, 31°11'43"N, 93°59'11"E, 4–5.x.2024, 4045 m (Chen Enyong, Yang Chengfeng and Chen Shuai leg.), unique IDs: TU-00866, 00868, 00869, 00871; • 1 ♀, Mira Mountain, Gaxing Township, Gongbu Jiangda County, Linzhi City, Xizang, 29°51'1.09"N, 92°20'27.55"E, 27.ix.2024, 4902.8 m (Chen Enyong leg.), unique ID: TU-00914; • 6 ♂♂, same data as previous but 29°50'30.22"N, 92°19'37.65"E, 12.ix.2024, 4275.9 m, unique IDs: TU-00991 to 00994, 00997, 00998.

###### Diagnosis.

The species largely varies in its forewing colouration (from mouse grey to ochreous with slate grey suffusion) and the degree of the forewing pattern development within the same population. *Dasypoliapolyformis* sp. nov. is most morphologically similar to *D.cerritula*, from which the new species differs in the somewhat larger size, the broader forewing, and the paler ground colour and cilia of both wings. In the male genital capsule, *D.polyformis* sp. nov. is distinguished from *D.cerritula* by the broader uncus, the somewhat narrower cucullus with thinner setae, the somewhat longer ampulla extending beyond the ventral margin of the valva, and the somewhat broader but shorter juxta with a narrower medio-dorsal process. As the female of *D.cerritula* is unknown, the female genitalia of *D.polyformis* sp. nov. were compared with *D.sejilaensis* and *D.vignai*, from which the new species differs in the narrower ostium bursae, the shorter anterior sclerotised plate of the ductus bursae, and the corpus bursae less curved sideways.

###### Description.

**External morphology of adults** (Figs 1–9). Forewing length 12.0–13.0 mm in males and 13.0–14.0 mm in females. Antenna serrulate in male and filiform in female. Body covered with long hair-like scales, dark brownish-grey with admixture of pale grey. Forewing elongate, with antemedially convex anal margin and evenly convex outer margin. Forewing ground colour varying from mouse grey to ochreous with grey suffusion. Forewing pattern diffuse, blackish-brown, sometimes indistinct. Subbasal line short, indistinct. Subbasal longitudinal dash narrow, diffuse. Antemedial line irregularly sinuous. Orbicular marking elliptical, pale with blackish-brown margin. Reniform marking narrow, semilunar, pale with indistinct dark margins. Postmedial line antero-medially curved outwards, dentate on veins. Subterminal line interrupted into row of blackish irregular spots of various sizes. Terminal line black, interrupted into spots between veins. Forewing cilia long, greyish-brown. Hindwing pale grey, suffused with brownish grey, with thin greyish-brown marginal line and large and diffuse, semilunar grey discal spot. Hindwing cilia long, pale brownish-grey. **Male genitalia** (Figs 39–41). Tegumen short, penicular lobe large, trapezoid with elongate posterior corner. Vinculum somewhat longer than tegumen, robust, U-shaped. Valva lobular with well-sclerotised costa and oblique editum bearing digitiform and apically pointed distal ampulla directed distally-ventrally and protruding beyond the ventral margin of valvula and exceeding ventral corner of cucullus. Cucullus trapezoidal with rounded corners, densely covered with spine-like setae. Sacculus broad (~2/3 of valva width proximally). Clasper oblique, slightly curved and dilated distally, without harpe. Valvula shortly triangular, slightly protruding ventrally. Juxta heavily sclerotised, broad, rectangular with rounded corners, with short, triangular, and apically pointed dorso-medial process. Phallus broad with rounded coecum, distally dilated. Proximal section of vesica granulose, as broad as distal end of phallus, distally tapered and extended into membranous vesica ejaculatorius directed distally, with short semiglobular dorsal subbasal diverticulum, and two unequally elongate longitudinal clusters of spike-like cornuti on its sides. **Female genitalia** (Figs 59, 60). Ovipositor short, broad, conical. Papilla analis elongate trapezoidal with rounded corners, weakly sclerotised and setose. Apophyses elongate and narrow, rod-like, heavily sclerotised, slightly tapered distally, anterior one shorter than posterior one (~70% of its length). Ostium bursae broad, its ventral margin with heavily sclerotised, belt-like antevaginal plate. Ductus bursae asymmetrical with somewhat longer right side and asymmetrical, sclerotised plates dilated to the right side: short and belt-like medial one, and broad and almost triangular anterior one. Posterior section of corpus bursae more or less tubular, somewhat dilated posteriorly. Anterior section of corpus bursae broad, teardrop-shaped, its ventral wall medio-posteriorly with small signum with irregular margins varying in size and degree of sclerotisation. Appendix bursae vestigial, conical, situated postero-laterally on right side at corner of anterior sclerotised plate of ductus bursae.

###### Etymology.

The specific epithet is an adjective derived from the Greek *πολυφόρμησ* (polyphormis) meaning polymorphic and refers to the fact that the new species has several colour forms.

###### Distribution.

The new species is currently known from three localities in southern Xizang, China.

##### ﻿ Dasypolia (Tatsipolia) cerritula

Taxon classificationAnimaliaLepidopteraNoctuidae

Chen, Pan, Volynkin, Saldaitis & Benedek, 2022

BA24114A-0ACA-5167-A3C9-349D954A3B36

Figs 10 , 42

Dasypolia (Tatsipolia) cerritula Chen et al., 2022: 192, figs 4, 11 (type locality: “Sejila Mountain, Linzhi City, Xizang, China, N:29°37'2" E:94°38'30" ... 4500 m”).

###### Type material examined.

***Holotype*** (Figs 10, 42): China • ♂, “STS-32788 | Sejila Mountain, Linzhi City, | Xizang, China, | N:29°37'2" | E:94°38'30" | 4-X-2020 | h [Altitude] 4500 m (coll. [leg.:] Pan Zhaohui | and Chen Enyong)” (TAAHU).

###### Diagnosis.

The forewing length is 11.5 mm in the male holotype. *Dasypoliacerritula* is most morphologically similar to *D.polyformis* sp. nov. and a detailed comparison is provided above in the diagnosis of the latter. Another similar species is the sympatric *D.sejilaensis*, from which *D.cerritula* can be distinguished by the greyish-brown hindwing with a smaller and rounded discal spot whereas the hindwing of *D.sejilaensis* is creamy with intense greyish suffusion outwardly and along the costal and anal margins, and its discal spot is large and falcate. The abdomen of *D.cerritula* is covered with black hair-like scales medially and distally whereas it is unicolourous brown in *D.sejilaensis*. In the male genital capsule, *D.cerritula* differs from *D.sejilaensis* in the narrower uncus, the larger penicular lobe with a more elongated posterior corner, and the shorter valva with a markedly broader cucullus densely covered with more robust setae. Additionally, the ampulla of *D.cerritula* is shorter and thinner than in *D.sejilaensis*, the valvula is markedly shorter and not protruding ventrally, the harpe is absent (it is present and tubercle-like in *D.sejilaensis*), and the juxta is narrower and bears a somewhat shorter and basally broader posterior medial process. The phalli and the vesica configurations of the two species are very similar but *D.cerritula* has two clusters of cornuti (vs 1 in *D.sejilaensis*).

###### Distribution.

The species is currently known only from its type locality in southern Xizang province of China.

##### ﻿ Dasypolia (Tatsipolia) sejilaensis

Taxon classificationAnimaliaLepidopteraNoctuidae

Chen, Pan, Volynkin, Saldaitis & Benedek, 2022

33F56992-3F58-5B6E-BFC6-8D40FC8E4BBF

**Figures 11–20.**
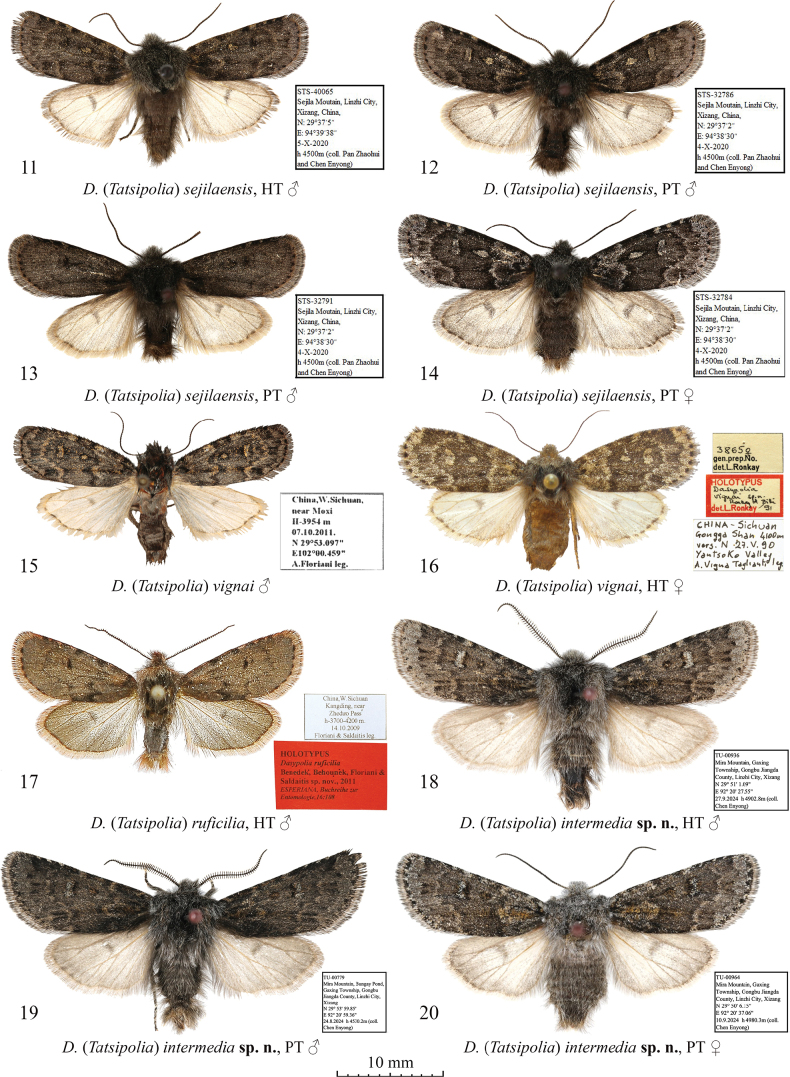
Adults of Dasypolia (Tatsipolia) spp. Depositories of the specimens: **11–14.** In TAAHU; **15.** In AFM; **16.** In HNHM (photo by B. Tóth); **17.** In ZSM; **18–20.** In TU.

Figs 11–14 , 43 , 61

Dasypolia (Tatsipolia) sejilaensis Chen et al. 2022: 189, figs 1–3, 9, 10, 15 (type locality: “Sejila Mountain, Linzhi City, Xizang, China, N:29°37'5" E:94°39'38" ... 4500 m”).

###### Type material examined.

***Holotype*** (Figs 11, 43): China • ♂, “STS-40065 | Sejila Mountain, Linzhi City, | Xizang, China, | N:29°37'5" | E:94°39'38" | 5-X-2020 | h [Altitude] 4500 m (coll. [leg.:] Pan Zhaohui | and Chen Enyong)” (TAAHU). ***Paratypes*.** China • 5 ♂♂, 1 ♀, Sejila Mountain, Linzhi City, Xizang, China, 29°37'2"N, 94°38'30"E, 4-X-2020, h [Altitude] 4500 m (Pan Zhaohui and Chen Enyong leg.), unique IDs: STS-32784, 32786, 32789 to 32792 (TAAHU).

###### Diagnosis.

The forewing length is 11.0–12.0 mm in males and 13.0 mm in the female. *Dasypoliasejilaensis* is externally reminiscent of *D.vignai* but is distinguished by its forewing shape, which has a straight costal margin and a more elongate apex, and the more diffuse forewing pattern in males, and the longer hindwing discal spot in both sexes. Additionally, compared to *D.vignai*, the reniform stigma of *D.sejilaensis* is situated closer to the forewing costa, and the pale suffusion on the transverse lines and stigmata is grey whereas it is brown in the congener. The male genital capsule of *D.sejilaensis* differs from *D.vignai* in the broader valva with a broader and less downcurved cucullus, the shorter but markedly thicker and upcurved ampulla (it is downcurved in *D.vignai*), the broader sacculus, and the less prominent and triangular valvula, which is more rounded in *D.vignai*. Additionally, the uncus, penicular lobe and juxta of *D.sejilaensis* are broader than in *D.vignai*. The phallus of *D.sejilaensis* is shorter and broader than in *D.vignai* (in proportion to the genital capsule). The vesicae of the two species are similar but the cornuti are more or less equal in size in *D.sejilaensis* whereas the distal cornuti of *D.vignai* are markedly longer and thicker than the proximal ones. In the female genitalia, *D.sejilaensis* can be distinguished from *D.vignai* in the longer apophysis anterior (in proportion to the ovipositor length), the narrower, more asymmetrically sclerotised and sideways curved ductus bursae (it is nearly straight in *D.vignai*), and the straight posterior section of the corpus bursae, which is sideways curved in *D.vignai*. A detailed comparison with another similar species, the sympatric *D.cerritula* is provided above in the diagnosis of the latter.

###### Distribution.

The species is currently known only from its type locality in southern Xizang province of China.

##### ﻿ Dasypolia (Tatsipolia) vignai

Taxon classificationAnimaliaLepidopteraNoctuidae

L. Ronkay & Zilli, 1992

5F83432C-A7EB-573F-A687-73337E583DC8

Figs 15 , 16 , 44 , 62

Dasypolia (Sinipolia) vignai Ronkay & Zilli, 1992: 500, fig. 12 (female genitalia), pl. O: fig. 11 (adult) (type locality: “China – Sichuan, Gongga Shan 4100 m ... Yantsoko Valley”).

###### Type material examined.

***Holotype*** (Figs 16, 62): • ♀, “China – Sichuan | Gongga Shan 4100 m | vers. N 27.V.[19]90 | Yantso Ko Valley, A.Vigna Taglianti leg.” | “Holotypus | *Dasypolia* | *vignai* sp.n. | Ronkay & Zilli | det. L.Ronkay/91" / “3865♀ | gen.prep.No. | det.L.Ronkay” (HNHM).

###### Additional material examined.

China • 1 ♂, W Sichuan, near Moxi, 3954 m, 07.x.2011, 29°53.097'’N, 102°00.459'’E, A. Floriani leg., gen. slide no.: JB2170 (AFM).

###### Diagnosis.

The forewing length is 12.0 mm in both sexes. *Dasypoliavignai* is externally similar to *D.sejilaensis* and *D.cerritula* but distinguished by the forewing having a slightly convex costal margin and a shorter and more rounded apex, and the more distinct forewing pattern in males, and the shorter hindwing discal spot in both sexes. Additionally, compared to the similar congeners, in *D.vignai* the reniform stigma is situated more inwardly from the forewing costa. The male genitalia of *D.vignai* differ clearly from other species in the *D.vignai* species group in the narrow cucullus, the long, downcurved and apically pointed ampulla, and the large and rounded valvula. A detailed comparison with the most morphologically similar *D.sejilaensis* is provided above in the diagnosis of the latter.

###### Distribution.

The species is known from two localities in Sichuan Province, south-western China.

##### ﻿ Dasypolia (Tatsipolia) ruficilia

Taxon classificationAnimaliaLepidopteraNoctuidae

Benedek, Behounek, Floriani & Saldaitis, 2011

B3A43BA7-6E8E-57EF-A31F-50AB1B4607CB

Figs 17 , 45

Dasypolia (Tatsipolia) ruficilia Benedek et al., 2011: 108, fig. 2 (male genitalia), pl. 15: fig. 4 (adult) (type locality: “China, W. Sichuan, Kangding, near Zheduo Pass, 3700–4200 m”).

###### Type material examined.

***Holotype*** (Figs 17, 45): • ♂, “China, W.Sichuan | Kangding, near | Zheduo Pass | h-3700–4200 m. | 14.10.2009 | Floriani & Saldaitis leg.” / red label “Holotypus | *Dasypoliaruficilia* | Benedek, Behounek, Floriani & | Saldaitis sp. nov., 2011 | *Esperiana*, Buchreihe zur | Entomologie, 16: 108,” gen. prep. No.: BG7015 (prepared by Behounek) (ZSM).

###### Diagnosis.

The forewing length is 12.0 mm in the male holotype. *Dasypoliaruficilia* differs externally from other species in the *D.vignai* species group in the unicolourous pale brown forewing ground colour and the reddish-brown forewing cilia, and is most reminiscent of *D.brandstetteri*, from which, however, it can be easily distinguished by the markedly shorter rami of the male antenna and the lack of the longitudinal black stripes in the cell and along the vein A1, which are characteristic of *D.brandstetteri*. Additionally, unlike in *D.brandstetteri*, *D.ruficilia* has a forewing with a more medially convex costal margin and a less elongate apex, reddish-brown cilia, and distinct transverse lines. The male genitalia structure of *D.ruficilia* is similar to *D.intermedia* sp. nov. and a detailed comparison is provided below in the diagnosis of the latter.

The female is unknown.

###### Distribution.

The species is known only from its type locality in western Sichuan Province of China (Benedek et al. 2011).

##### ﻿ Dasypolia (Tatsipolia) intermedia sp. nov.

Taxon classificationAnimaliaLepidopteraNoctuidae

569D9245-B772-5028-9431-977FCFEA1749

https://zoobank.org/18988E0B-DF4F-4366-85C4-A1288D0645F3

Figs 18–20 , 46 , 47–48 , 63

###### Type material.

***Holotype*** (Figs 18, 46): China • ♂, “TU-00936 | Mira Mountain, Gaxing | Township, Gongbu Jiangda | County, Linzhi City, Xizang | N 29° 51' 1.09" | E 92° 20' 27.55" | 27.9[ix].2024 h [altitude] 4902.8 m (coll. [leg.:] | Chen Enyong)” (TU). ***Paratypes*** (3 ♂♂, 1 ♀, all in TU). China • 1 ♂, same data as in holotype, unique ID: TU-00935; • 1 ♀, same data as previous but 29°50'6.15"N, 92°20'37.06"E, 10.ix.2024, 4980.3 m, unique ID: TU-00964; • 2 ♂♂, Mira Mountain, Bungay Pond, Gaxing Township, Gongbu Jiangda County, Linzhi City, Xizang, 29°53'59.85"N, 92°20'59.36"E, 24.viii.2024, 4530.2 m (Chen Enyong leg.), unique IDs: TU-00779, 00892.

###### Diagnosis.

*Dasypoliaintermedia* sp. nov., with its larger size, elongate forewing and bipectinate male antenna, is externally readily different from other species in the *D.vignai* species group and is reminiscent of certain members of the *D.nivalis* species group, namely *D.diffusa* sp. nov., from which *D.intermedia* sp. nov. is distinguished by the more elongate forewing with a darker ground colour and more distinct markings. The male genital capsule of the new species is most reminiscent of the externally dissimilar *D.ruficilia* but differs in the narrower uncus, the longer and broader valva, the shorter cucullus with a narrower setose area, the thicker ampulla directed more distally, and the broader clasper with a slightly larger harpe directed anteriorly (inwards). Additionally, unlike *D.ruficilia*, the juxta of *D.diffusa* sp. nov. is more weakly sclerotised, anteriorly narrower, and bears a shorter and triangular medio-dorsal process, which is thumb-shaped in *D.ruficilia*. The phallus of *D.diffusa* sp. nov. is distally narrower than in *D.ruficilia*, and the vesica has a considerably longer proximal section and bears only one cluster of larger cornuti whereas there are two clusters in *D.ruficilia*. As the female of *D.ruficilia* is unknown, the female genitalia of *D.diffusa* sp. nov. were compared with other species of *Tatsipolia* s. str., from which the new species differs clearly in the markedly longer posterior sclerotised region of the ductus bursae forming a glass-shaped antrum (whereas in other species of the *D.vignai* species group, it is short and belt-like), and the gelatinous and globular anterior section of the corpus bursae bearing a sclerotised plate, which is similar to that of certain species of the *D.nivalis* species group, whereas in other species in the *D.vignai* species group, the anterior section of the corpus bursae bears a larger and flattened sclerotised plate. Additionally, the anterior section of the corpus bursae of the new species lacks the signum, which is present in other members of the *D.vignai* species group but also absent in the *D.nivalis* species group.

###### Description.

**External morphology of adults** (Figs 18–20). Forewing length 15.0 mm in males and 14.5 mm in female. Antenna bipectinate in male and filiform in female. Body brownish grey with admixture of pale grey. Forewing elongate and narrow, with convex outer margin and rounded apex. Forewing ground colour slate grey, darker in medial area. Forewing pattern diffuse, blackish-grey. Basal line branchy, black, short. Antemedial line irregularly sinuous, edged with pale grey along inwardly. Claviform marking strongly elongate and protruding into medial area, diffuse ochreous yellow. Orbicular marking small, circular. Reniform marking with blackish margins and filled with off-white sparsely suffused with grey scales. Costal margin with series of small pale grey spots. Postmedial line smoothly question mark-like curved, shortly dentate on veins, edged with pale grey outwardly. Subterminal line irregular, interrupted into triangular spots of different sizes. Subterminal area with intense pale grey suffusion. Terminal line interrupted into small and irregular blackish spots between veins. Forewing cilia pale grey. Hindwing pale brownish-grey with weak grey suffusion along costal and outer margins. Discal spot grey, broad semilunar, diffuse. Hindwing cilia pale ochreous grey. **Male genitalia** (Figs 46–48). Uncus short, triangular, dorso-ventrally swollen, distally tapered, and apically pointed and downcurved. Tegumen short with swollen and semiglobular penicular lobes. Vinculum longer than tegumen, robust, U-shaped. Valva longer than annulus, Proximally upcurved and with straight distal section with almost parallel margins. Costa narrow and moderately sclerotised, distally reaching cucullus. Cucullus short, rounded, densely covered with setae. Editum long, stretching along costa but distally evenly diverging from it ventrally, with digitiform and apically rounded distal ampulla protruding beyond ventral margin of valva below cucullus ventral edge. Sacculus ~1/2 of valva width, somewhat tapered distally. Clasper oblique, almost straight, dorsally dilated and bearing short tubercle-like harpe directed proximally. Valvula elongate and not protruding ventrally. Juxta trapezoidal, with conical and apically rounded medio-dorsal process. Phallus cylindrical, medially somewhat downcurved, with dilated and rounded coecum. Proximal section of vesica weakly granulose, tubular, proximally as broad as phallus but distally dilated. Distal section of vesica more or less globular and dorsally bearing bunch-like cluster of spine-like cornuti of different sizes. Vesica ejaculatorius membranous, originating apically and directed distally-dorsally. **Female genitalia** (Fig. 63). Ovipositor broad and conical. Papilla analis short, trapezoidal, weakly sclerotised, setose. Apophyses elongate and thin but well-sclerotised, rod-like; anterior one slightly longer than posterior one. Ostium bursae broad, with concave ventral margin. Ductus bursae elongate, its posterior sclerotised region large and transformed into glass-shaped antrum. Medial section of ductus bursae short and membranous. Anterior section of ductus bursae tapered posteriorly, its posterior part with weak medial sclerotisation while anterior part dilated, globular, gelatinous with sclerotised plate anterio-laterally. Corpus bursae egg-shaped, membranous, shorter than ductus bursae. Appendix bursae vestigial.

###### Etymology.

The specific epithet is a Latin adjective meaning intermediate and refers to the external and genital morphology of the new species, which appears as intermediate between the *D.vignai* and *D.nivalis* species groups.

###### Distribution.

The new species is currently known from its type locality in southern Xizang, China.

#### ﻿The *D.nivalis* species group

**Diagnosis.** The male genitalia of the *D.nivalis* species group differ from the *D.vignai* species group in the well-developed, clavate harpe. In the female genitalia, compared to the *D.vignai* species group, the ostium bursae is considerably narrower, the ductus bursae has two regions of sclerotisation with a membranous ventral margin of the ostium bursae, and the anterior section of the corpus bursae is longer than the ovipositor.

##### ﻿ Dasypolia (Tatsipolia) brandstetteri

Taxon classificationAnimaliaLepidopteraNoctuidae

Benedek, Behounek, Floriani & Saldaitis, 2011

8293B860-FB27-5E1C-88F6-A84BBBF96129

Figs 21 , 49

Dasypolia (Tatsipolia) brandstetteri Benedek et al., 2011: 109, fig. 2 (male genitalia), pl. 15: figs 5, 6 (adults) (type locality: “China, W. Sichuan, Kangding, near Zheduo Pass, 3700–4200 m”).

###### Type material examined.

**Figures 21–28.**
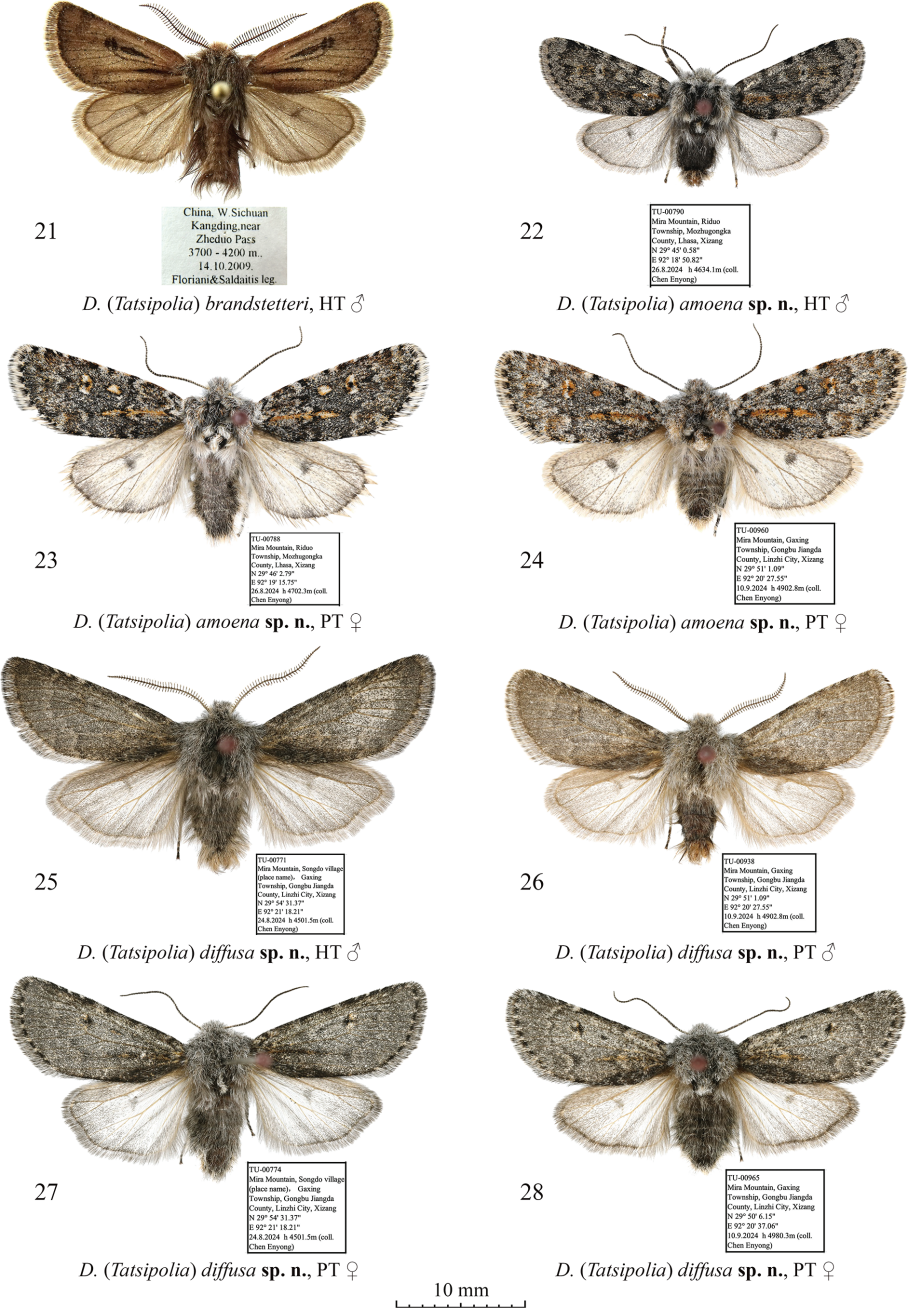
Adults of Dasypolia (Tatsipolia) spp. Depositories of the specimens: **21.** In ZSM; **22–28.** In TU.

***Holotype*** (Figs 21, 49): • ♂, “China, W.Sichuan | Kangding near | Zheduo Pass | 3700–4200 m., | 14.10[x].2009. | Floriani&Saldaitis leg.”, gen. slide. No.: JB1477 (ZSM). ***Paratypes*.** China • 4 ♂♂, same data as in holotype (AFM, ASV, BBT).

###### Diagnosis.

The forewing length is 12.0 mm in males. *Dasypoliabrandstetteri* with its strongly bipectinate male antenna, brown colouration, and the lack of the forewing transverse lines, is externally dissimilar to other species in the *D.nivalis* species group and is most reminiscent of *D.ruficilia*, a detailed comparison with which is provided above in the diagnosis of the latter. The male genital capsule of *D.brandstetteri* is distinct from other species in the *D.nivalis* species group by the narrower cucullus and the triangular valvula protruding ventrally. The vesica structure of *D.brandstetteri* is most reminiscent of *D.nivalis* with its vesica having a ventral diverticulum but in the former species the diverticulum is longer and bilobate.

The female is unknown.

###### Distribution.

The species is currently known only from its type locality in western Sichuan Province, China.

##### ﻿ Dasypolia (Tatsipolia) amoena sp. nov.

Taxon classificationAnimaliaLepidopteraNoctuidae

66D6A9A2-6A7D-5A69-9F0C-F956AD6593F2

https://zoobank.org/6C6C5D4E-16D0-4E23-928E-8F3CFDA9ED12

Figs 22–24 , 50 , 64

###### Type material.

***Holotype*** (Figs 22, 50): China • ♂, “TU-00790 | Mira Mountain, Riduo | Township, Mozhugongka | County, Lhasa, Xizang | N 29°45'0.58" | E 92°18' 50.82" | 26.8[viii].2024 h [altitude] 4634.1 m (coll. [leg.:] | Chen Enyong)” (TU). ***Paratypes*** (2 ♀♀, in TU). China • 1 ♀, same data as in holotype but 29°46'2.79"N, 92°19'15.75"E, 4702.3 m, unique ID: TU-00788; • 1 ♀, Mira Mountain, Gaxing Township, Gongbu Jiangda County, Linzhi City, Xizang, 29°51'1.09"N, 92°20'27.55"E, 27.ix.2024, 4902.8 m (Chen Enyong leg.), unique ID: TU-00960.

###### Diagnosis.

*Dasypoliaamoena* sp. nov. is externally vaguely reminiscent of *D.nivalis* but can be readily distinguished by the serrulate male antenna (it is shortly bipectinate in *D.nivalis*), the somewhat broader forewing with slightly more convex costal and anal margins, the bluish-grey forewing ground colour (vs brown or grey brown in *D.nivalis*), the orange suffusion on the claviform, orbicular and reniform markings and the subterminal line, and the lack of the intense grey suffusion along the hindwing costal and outer margins, which is characteristic of *D.nivalis*. Additionally, the head and the thorax of the new species are pale grey with admixture of dark grey and ochreous hair-like scales whereas in *D.nivalis* they are more unicolourous dark brown. The male genital capsule of *D.amoena* sp. nov. is similar to *D.diffusa* sp. nov., *D.luxuriosa* sp. nov., *D.ultramontana* sp. nov. and *D.nivalis* but differs in the broader cucullus and the apically narrower harpe. The phallus of *D.amoena* sp. nov. is shorter and broader than in the similar species. The vesica configuration of *D.amoena* sp. nov. is most similar to *D.ultramontana* sp. nov. with its semiglobular ventral diverticulum but differs in the markedly smaller cornuti gathered into two more elongate clusters, which are more distant from each other than in the congener. Among species of the *D.nivalis* species group with known females, the female genitalia of *D.luxuriosa* sp. nov. are most similar to *D.amoena* sp. nov., but in the latter the medial, membranous section of the ductus bursae is longer, the sclerotised plates of the anterior gelatinous region of the ductus bursae are larger, and the corpus bursae is considerably shorter.

###### Description.

**External morphology of adults** (Figs 22–24). Forewing length 11.0 mm in male holotype and 14.0 mm in females. Female larger than male and with more elongate forewing, paler ground colour and more distinct and contrast pattern, and more intense orange suffusion on certain markings. Antenna shortly serrulate in male and filiform in female. Body pale grey with admixture of blackish scales. Forewing elongate with oblique tornus. Forewing ground colour grey with blackish suffusion. Pattern elements dark grey. Subbasal and antemedial lines irregularly sinuous. Costa with irregular blackish spots. Claviform dash elongate and protruding into medial area, suffused with orange. Orbicular marking small, elliptical with diffuse blackish margins, filled with pale grey in male and suffused with orange in female. Reniform marking pale grey with diffuse blackish margins and black dot-like core spot, in female intensely suffused with orange proximally and distally. Postmedial line thin, question mark-like curved, dentate on veins, suffused with pale grey along outer margin. Subterminal line irregular and diffuse, in female suffused with orange. Terminal line black, interrupted on veins. Forewing cilia pale grey with admixture of dark grey. Hindwing pale grey with dark grey suffusion, darker in male. Terminal line dark brownish-grey, thin. Discal spot elliptical and diffuse, broader in female. Hindwing cilia pale ochreous. **Male genitalia** (Fig. 50). Uncus elongate, distally tapered, and apically pointed. Tegumen with short and swollen, rounded penicular lobes. Vinculum as long as tegumen, heavily sclerotised, U-shaped. Valva lobular, somewhat shorter than annulus, with almost parallel margins. Costa narrow, distally dilated and reaching cucullus. Cucullus lobular, rounded, densely covered with robust setae. Editum thin and weakly sclerotised, stretching along costa and fused with it dorsally, bearing short triangular and apically rounded, ventrally directed distal ampulla originating at the ventral corner of cucullus and not protruding beyond the ventral margin of valvula. Valvula short and not protruding ventrally. Sacculus trapezoidal, broad (~3/4 of valva width). Clasper oblique, straight, apically dilated, with elongate clavate harpe well-protruding beyond dorsal margin of valva. Juxta trapezoidal, with short, triangular medio-dorsal process. Phallus broad with rounded coecum, somewhat dilated distally. Proximal section of vesica short and somewhat broader than phallus, with very short but broad, protrusion-like ventral diverticulum and two short longitudinal clusters of small spine-like cornuti dorsally and ventrally. Vesica ejaculatorius tubular and membranous, originating apically and directed distally. **Female genitalia** (Fig. 64). Ovipositor short, conical. Papilla analis elliptical, setose. Apophyses elongate and thin, proximally flattened and distally rod-like, more or less equal in length. Ostium bursae moderately broad, with membranous margins. Ductus bursae elongate, somewhat constricted medially and globular and gelatinous anteriorly. Medial sclerotised plate situated posteriorly, elliptical. Anterior gelatinous section of ductus bursae with two medio-lateral sclerotised plates of different sizes. Corpus bursae membranous, elongate pear-shaped. Appendix bursae vestigial, conical, situated ventrally at junction with ductus bursae.

###### Etymology.

The specific epithet is a Latin adjective meaning pleasant and refers to the beautiful external appearance of the new species.

###### Distribution.

The new species is currently known from two localities in southern Xizang, China.

##### ﻿ Dasypolia (Tatsipolia) diffusa sp. nov.

Taxon classificationAnimaliaLepidopteraNoctuidae

C22F0F88-863A-5786-B197-D5EB980573E1

https://zoobank.org/F5D61C2E-AEAA-49D6-AF84-3C372C8D198C

Figs 25–28 , 51 , 52 , 65

###### Type material.

***Holotype*** (Figs 25, 51): China • ♂, “TU-00771 | Mira Mountain, Songdo village | (place name), Gaxing | Township, Gongbu Jiangda | County, Linzhi City, Xizang | N 29° 54' 31.37" | E 92° 21' 18.21" | 24.8[viii].2024 h [altitude] 4501.5 m (coll. [leg.:] | Chen Enyong)” (TU). ***Paratypes*** (5 ♂♂, 4 ♀♀, all in TU). China • 2 ♂♂, 1 ♀, same data as in holotype, unique IDs: TU-00772–00774; • 1 ♀, same data as previous but 29°53'1.70"N, 92°31'1.18"E, 26.ix.2024, 4236.6 m (Chen Enyong leg.), unique ID: TU-00889; • 2 ♂♂, 1 ♀, same data as previous but 29°51'1.09"N, 92°20'27.55"E, 10.ix.2024, 4902.8 m (Chen Enyong leg.), unique IDs: TU-00937–00939; • 1 ♀, same data as previous but 29°50'6.15"N, 92°20'37.06"E, 10.ix.2024, 4980.3 m (Chen Enyong leg.), unique ID: TU-00965; • 1 ♂, Mira Mountain, Bungay Pond, Gaxing Township, Gongbu Jiangda County, Linzhi City, Xizang, 29°53'59.85"N, 92°20'59.36"E, 24.viii.2024, 4530.2 m (Chen Enyong leg.), unique ID: TU-00893.

###### Diagnosis.

*Dasypoliadiffusa* sp. nov. is externally clearly different from other species in the *D.nivalis* species group by its ash-grey forewing ground colour and the indistinct forewing markings. The male genital capsule structure of *D.diffusa* sp. nov. is most similar to *D.luxuriosa* sp. nov. but in the former the uncus is broader, the harpe is apically narrower, and the valvula is more protruding ventrally than in *D.luxuriosa* sp. nov. Compared to *D.luxuriosa* sp. nov., the vesica of *D.diffusa* sp. nov. has a markedly narrower proximal section and bears two elongate row-like longitudinal clusters of smaller cornuti whereas in *D.luxuriosa* sp. nov. the cornuti are gathered into short, bunch-like clusters. In the female genitalia, *D.diffusa* sp. nov. differs clearly from other species in the *D.nivalis* species group in the shorter ductus bursae with small and weakly sclerotised plates and lacking the gelatinous anterior dilation, and the shorter and broader corpus bursae.

###### Description.

**External morphology of adults** (Figs 25–28). Forewing length 14.0–15.5 mm in males and 14.0–15.0 mm in females. Antenna bipectinate in male and filiform in female. Body grey with admixture of pale grey scales. Forewing triangular with somewhat elongate and rounded apex. Forewing ground colour dark grey. Markings dark grey, diffuse more distinct in female. Antemedial line sinuous, edged with pale grey inwardly. Orbicular marking small, dot-shaped. Reniform marking pale grey with indistinct margins and dark grey core spot. Postmedial line question mark-like curved, slightly dentate on veins. Subterminal line irregular and indistinct. Terminal line black and interrupted on veins. Forewing cilia dark grey with slight admixture of pale grey. Hindwing pale grey with weak dark brownish-grey suffusion along costal and outer margins. Discal spot semilunar and diffuse. Terminal line thin, dark brownish-grey. Hindwing cilia grey. **Male genitalia** (Figs 51, 52). Uncus short, dorso-ventrally flattened and apically blunt. Tegumen with broad and rounded, swollen penicular lobes. Vinculum longer than tegumen, heavily sclerotised, U-shaped with rounded saccus directed anteriorly. Valva lobular, distally tapered and slightly upcurved. Costa narrow, distally dilated and distally reaching cucullus. Cucullus rounded and densely covered with setae. Editum thin, stretching along ventral margin of costa and dorsally fused with its ventral margin, bearing short, triangular, and apically blunt distal ampulla directed distally-ventrally, reaching the ventral margin of valva but not protruding beyond it. Valvula short but broad, forming short ventral protrusion. Sacculus trapezoidal and broad (~3/4 of valva width). Clasper oblique, ventrally straight but dorsally dilated and slightly upcurved, bearing narrow clavate harpe slightly protruding beyond dorsal margin of valva. Juxta narrow, trapezoidal, with short, triangular medio-dorsal process. Phallus narrow and cylindrical, with broad and rounded coecum and narrower medial and distal sections. Vesica tubular, narrower than phallus, ventrally with two longitudinal clusters of minute, spine-like cornuti stretching parallel to each other. Vesica ejaculatorius narrow, tubular, directed distally. **Female genitalia** (Fig. 65). Ovipositor short and conical. Papilla analis elliptical and setose. Apophyses elongate and thin, rod-like, equal in length. Ostium bursae narrow with membranous margins. Ductus bursae narrow, with short and weakly sclerotised plate posteriorly and broader, elliptical sclerotised plate anteriorly. Corpus bursae membranous, pear-shaped with constricted posterior section. Appendix bursae vestigial, conical, situated postero-ventrally at anterior edge of anterior sclerotised plate of ductus bursae.

###### Etymology.

The specific epithet is a Latin adjective meaning diffuse and refers to the indistinct forewing pattern of the new species.

###### Distribution.

The new species is currently known from two localities in southern Xizang, China.

##### ﻿ Dasypolia (Tatsipolia) luxuriosa sp. nov.

Taxon classificationAnimaliaLepidopteraNoctuidae

D25D2891-2DCC-50DD-980B-D16842BF018E

https://zoobank.org/53A1AA13-5649-434E-88C6-97716B0DAF9A

Figs 29–34 , 53 , 54 , 66

###### Type material.

***Holotype*** (Figs 26, 53): China • ♂, “TU-00927 | Mira Mountain, Gaxing | Township, Gongbu Jiangda | County, Linzhi City, Xizang | N 29° 51' 1.09" | E 92° 20' 27.55" | 10.9[ix].2024 h 4902.8 m (coll. [leg.:] | Chen Enyong)” (TU). ***Paratypes*** (4 ♂♂, 8 ♀♀, all in TU). China • 3 ♀♀, same data as in holotype, unique ID: TU-00931–00933; • 1 ♀, same data as previous but 25.viii.2024, 4889.3 m, unique ID: TU-00786; • 1 ♂, 1 ♀, same data as previous but 29°53'59.85"N, 92°20'59.36"E, 24.viii.2024, 4530 m (Chen Enyong leg.), unique IDs: TU-00776, 00780; • 1 ♂, 3 ♀♀, same data as previous but 29°50'15.44"N, 92°19'26.12"E, 27.ix.2024, 4786.4 m, unique IDs: TU-00895, 00897, 00898, 00900; • 1 ♂, same data as previous but 10.xi.2024, unique ID: TU-00947; 1 ♂, same data as previous but 25.viii.2024, unique ID: TU-00891.

###### Diagnosis.

*Dasypolialuxuriosa* sp. nov. is externally vaguely reminiscent of *D.ultramontana* sp. nov. but differs in the shorter rami of the male antenna, the larger size, the broader forewing, the darker grey forewing ground colour, the less sinuous antemedial line, and the narrower, falcate reniform marking lacking the dark core spot. Additionally, unlike in *D.ultramontana* sp. nov., the inner triangular dashes of the subterminal line of *D.luxuriosa* sp. nov. are more distinct and longer, and the hindwing is suffused with brownish-grey medially and along the anal margin whereas in *D.ultramontana* sp. nov. it is paler but with intense grey suffusion along the costal and outer margins. The male genitalia of the two species are similar but in *D.luxuriosa* sp. nov. the uncus is somewhat longer, the juxta is broader, the sacculus is narrower, and the vesica lacks the ventral diverticulum. As the female of *D.ultramontana* sp. nov. is unknown, the female genitalia of *D.luxuriosa* sp. nov. were compared with *D.amoena* sp. nov., a detailed comparison with which is provided above in the diagnosis of the latter species.

###### Description.

**Figures 29–38.**
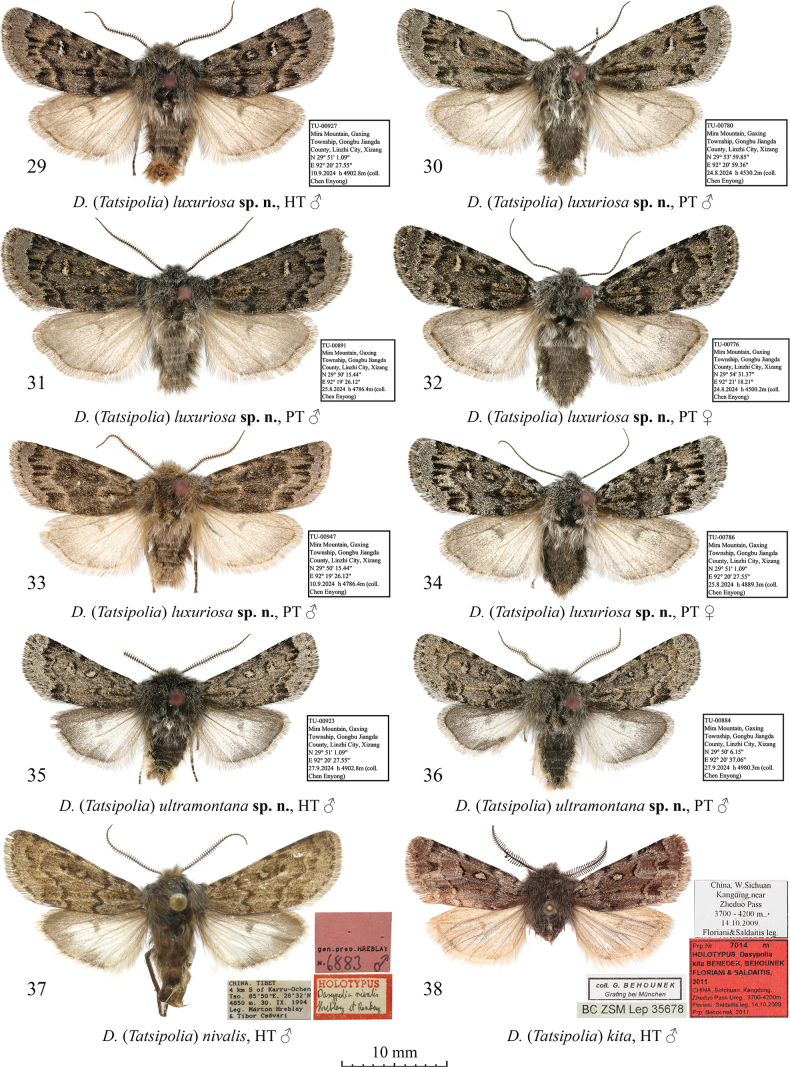
Adults of Dasypolia (Tatsipolia) spp. Depositories of the specimens: **29–36.** In TU; **37.** In MH/HNHM (photo by B. Tóth); **38.** In ZSM.

**External morphology of adults** (Figs 29–34). Forewing length 14.0–15.0 mm in males and 14.0 mm in females. Antenna shortly bipectinate in male and filiform in female. Head and thorax dark grey with admixture of pale grey. Forewing triangular with rounded apex and convex anal margin. Forewing ground colour grey with slight dark grey suffusion. Pattern elements blackish. Subbasal and antemedial lines irregularly sinuous, antemedial one edged with pale ochreous grey inwardly. Costa with series of irregular blackish spots. Orbicular marking elliptical, small, with blackish margins, filled with pale grey or off-white. Reniform marking semilunar, with blackish margins, filled with off-white. Postmedial line question mark-like curved, irregularly dentate on veins, edged with pale ochreous grey outwardly. Subterminal line irregular, interrupted into series of triangular dashes of different sizes. Terminal line thin, interrupted on veins. Cilia dark grey with admixture of pale grey scales. Hindwing pale ochreous grey with slight dark grey suffusion along costal and outer margins. Discal spot semilunar, diffuse. Terminal line thin, ochreous grey. Hindwing cilia grey. **Male genitalia** (Figs 53, 54). Uncus swollen, elongate, distally tapered and apically pointed. Tegumen with rounded and swollen penicular lobes. Vinculum somewhat longer than tegumen, heavily sclerotised, U-shaped. Valva lobular, slightly tapered distally. Costa distally dilated and reaching cucullus. Cucullus rounded and densely covered with setae. Editum thin, stretching along ventral margin of costa and dorsally fused with it, bearing narrow triangular distal ampulla directed ventrally and not protruding the ventral margin of valva. Sacculus trapezoidal, ~1/2 of valva width. Clasper oblique, ventrally straight and dorsally dilated and upcurved, bearing clavate harpe protruding beyond dorsal margin of valva. Valvula short but broad, not protruding ventrally. Juxta trapezoidal with short, triangular medio-dorsal process. Phallus cylindrical with rounded coecum, somewhat downcurved postmedially. Proximal section of vesica elliptical, broader than phallus, weakly granulose and bearing two bunch-like clusters of spine-like cornuti on lateral sides. Vesica ejaculatorius tubular and distally directed. **Female genitalia** (Fig. 66). Ovipositor broad and conical. Papilla analis trapezoidal with rounded corners, setose. Apophyses elongate and thin, rod-like, anterior one longer than posterior one. Ostium bursae moderately broad, with membranous margins. Posterior section of ductus bursae funnel-like dilated posteriorly, with medial rectangular sclerotised plate with postero-medial depression. Anterior section of ductus bursae irregularly globular, swollen, gelatinous with two rounded sclerotised plates of different sizes. Corpus bursae membranous, strongly elongate, with tubular posterior and pear-shaped anterior sections. Appendix bursae vestigial, ductus ejaculatorius originating posteriorly at junction with anterior gelatinous section of ductus bursae.

###### Etymology.

The specific epithet is a Latin adjective meaning luxurious and refers to the beautiful external appearance of the new species.

###### Distribution.

The new species is currently known from its type locality in southern Xizang, China.

##### ﻿ Dasypolia (Tatsipolia) ultramontana sp. nov.

Taxon classificationAnimaliaLepidopteraNoctuidae

48148C59-1DFC-51B8-B2A6-BA60C699854C

https://zoobank.org/9AAE2348-0421-4A5E-BA6D-5DE850021ADC

Figs 35 , 36 , 55 , 56

###### Type material.

***Holotype*** (Figs 35, 55): China • ♂, “TU-00923 | Mira Mountain, Gaxing | Township, Gongbu Jiangda | County, Linzhi City, Xizang | N 29° 51' 1.09" | E 92° 20' 27.55" | 10.9[ix].2024 h [altitude] 4902.8 m (coll. [leg.:] | Chen Enyong)” (TU). ***Paratype*.** China • ♂, same data as in holotype, unique ID: TU-00884 (TU).

###### Diagnosis.

*Dasypoliaultramontana* sp. nov. is morphologically similar to *D.nivalis*, from which the new species differs externally in the smaller size, the less elongate forewing with a less elongate apex and a more angular tornus, the brownish-grey body and forewing ground colour (vs pale brown in *D.nivalis*), the more zigzagged antemedial line, the smaller dark core of the reniform marking, and the less curved postmedial line. The male genital capsules of the two species are similar but in *D.ultramontana* sp. nov. the uncus is shorter and somewhat broader and the harpe is more distally dilated than in *D.nivalis*. In the vesica, the new species has two bunch-like clusters of cornuti (vs a single cluster in *D.nivalis*), and a shorter ventral diverticulum. A diagnostic comparison with another morphologically similar species, the sympatric *D.luxuriosa* sp. nov. is provided above in the diagnosis of the latter. The female is unknown.

###### Description.

**External morphology of adults** (Figs 35, 36). Forewing length 11.0–11.5 mm in males. Male antenna shortly bipectinate. Body dark brownish-grey with admixture of pale grey scales in head and thorax and intense admixture of brown in abdomen laterally and distally. Forewing elongate with almost straight costal and moderately convex anal and outer margins. Forewing ground colour brownish-grey with blackish-grey suffusion. Pattern elements blackish. Costal margin with series of irregular blackish spots. Subbasal line irregularly sinuous. Claviform marking dash-like, not reaching antemedial line, suffused with ochreous brown. Antemedial line irregularly zigzagged, edged with pale brownish-grey inwardly. Orbicular marking small, almost circular, filled with pale brownish-grey. Reniform marking filled with pale brownish-grey, with indistinct outer margin and small, vertical dash-shaped, blackish core spot. Postmedial line question mark-like curved, irregularly sinuous, edged with pale brownish-grey outwardly. Subterminal line irregular, edged with diffuse cuneal blackish spots of different sizes inwardly. Subterminal area pale grey with slight blackish suffusion. Terminal line interrupted into small spots on veins. Forewing cilia brownish-grey with admixture of blackish scales. Hindwing off-white medially, with intense brownish-grey suffusion along costal and outer margins, and slight brownish-grey suffusion along anal margin. Discal spot semilunar, brownish-grey, diffuse. Hindwing cilia pale brownish-grey. **Male genitalia** (Figs 55, 56). Uncus slightly swollen, rhomboidal, apically pointed. Tegumen with rounded and swollen penicular lobes. Vinculum somewhat longer than tegumen, heavily sclerotised, U-shaped, with short and rounded saccus. Valva lobular, tapered distally, with almost straight dorsal and convex ventral margins. Costa distally dilated and reaching cucullus. Cucullus rounded and densely covered with setae. Editum thin, stretching along ventral margin of costa and dorsally fused with it, bearing narrow triangular distal ampulla directed ventrally and reaching or slightly protruding beyond the ventral margin of valva. Sacculus trapezoidal, ~2/3 of valva width. Clasper oblique, ventrally straight and dorsally dilated and upcurved, bearing clavate harpe protruding beyond dorsal margin of valva. Valvula short but broad, not protruding ventrally. Juxta trapezoidal with thumb-shaped medio-dorsal process. Phallus cylindrical with rounded coecum, somewhat downcurved postmedially. Proximal section of vesica broader than phallus, proximally granulose and distally membranous, with short, semi-globular ventral diverticulum and two bunch-like clusters of short spine-like cornuti laterally and dorsally. Vesica ejaculatorius tubular and distally directed.

Female unknown.

###### Etymology.

The specific epithet is an adjective derived from the Latin *ultra* meaning super and *montanus* meaning mountainous, and refers to the occurrence of the new species at the highest altitudes.

###### Distribution.

The new species is currently known only from its type locality in southern Xizang, China.

##### ﻿ Dasypolia (Tatsipolia) nivalis

Taxon classificationAnimaliaLepidopteraNoctuidae

Hreblay & L. Ronkay, 1995

B8DB4F96-901F-5F6F-9E25-EB99D99AB469

Figs 37 , 57

Dasypolia (*s. l.*) nivalis Hreblay & Ronkay, 1995: 376, figs 30, 56, 57, 79 (type locality: “China, Tibet, 4 km S of Karru-Ochen Tso, 85°50'E, 28°32’N, 4850 m”).

###### Type material examined.

***Holotype*** (Figs 37, 57): ♂, “CHINA. TIBET | 4 km S of Karru-Ochen | Tso. 85°50’E, 28°32’N | 4850 m. 30. IX. 1994 | Leg. Márton Hreblay | & Tibor Csővári” / red framed label “HOLOTYPUS | Dasypolianivalis | Hreblay et Ronkay” / pink label “gen. prep. HREBLAY | N: 6883 ♂” (MH/HNHM).

**Figures 39–42.**
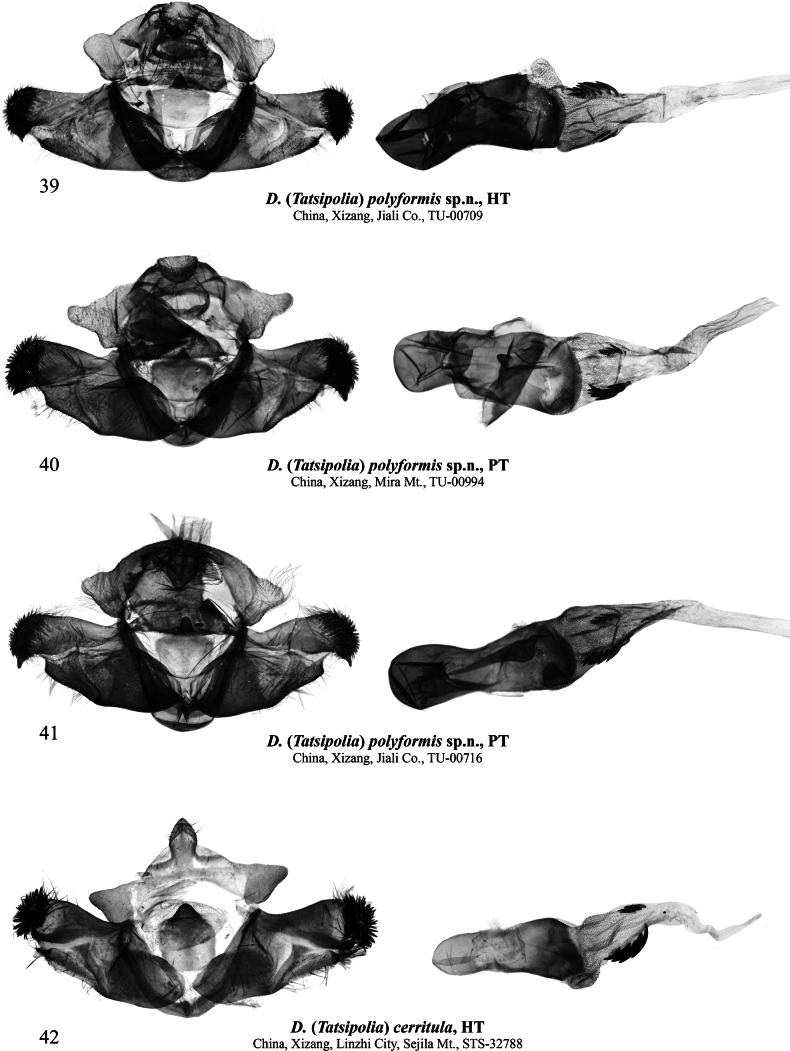
Male genitalia of Dasypolia (Tatsipolia) spp. Dissected of the specimens are deposited: **39–41.** In TU; **42.** In TAAHU.

**Figures 43–46.**
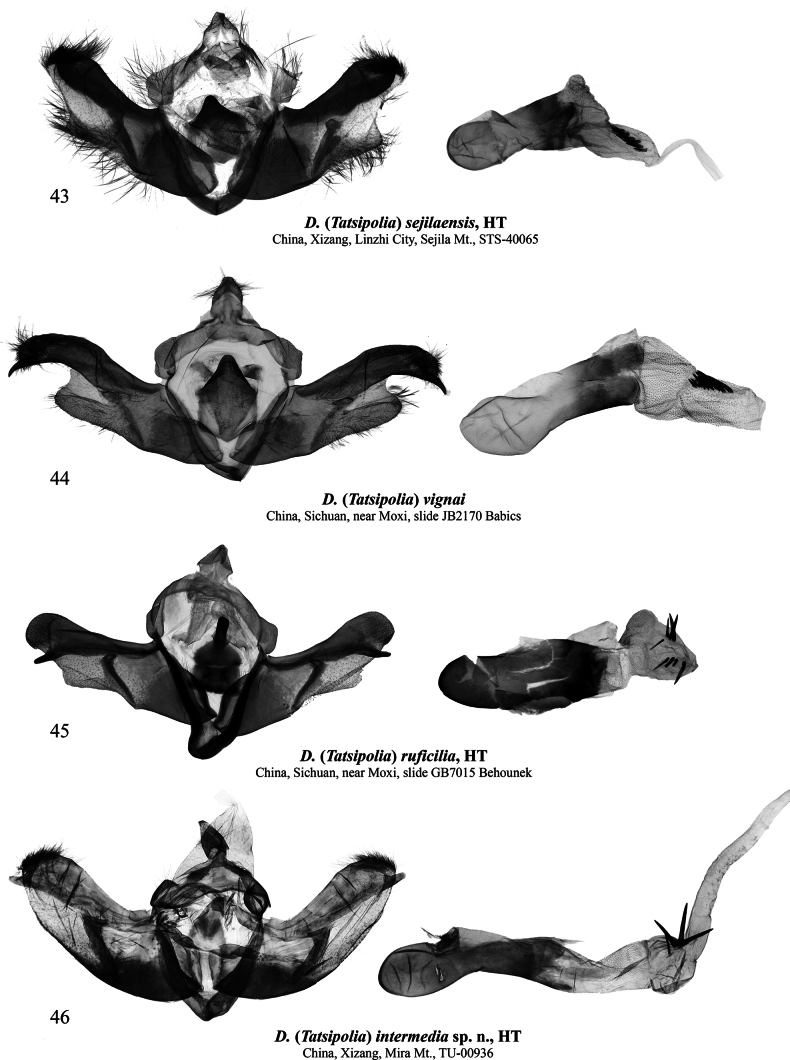
Male genitalia of Dasypolia (Tatsipolia) spp. Depositories of the specimens dissected: **43.** In TAAHU; **44.** In AFM; **45.** In ZSM; **46.** In TU.

**Figures 47–50.**
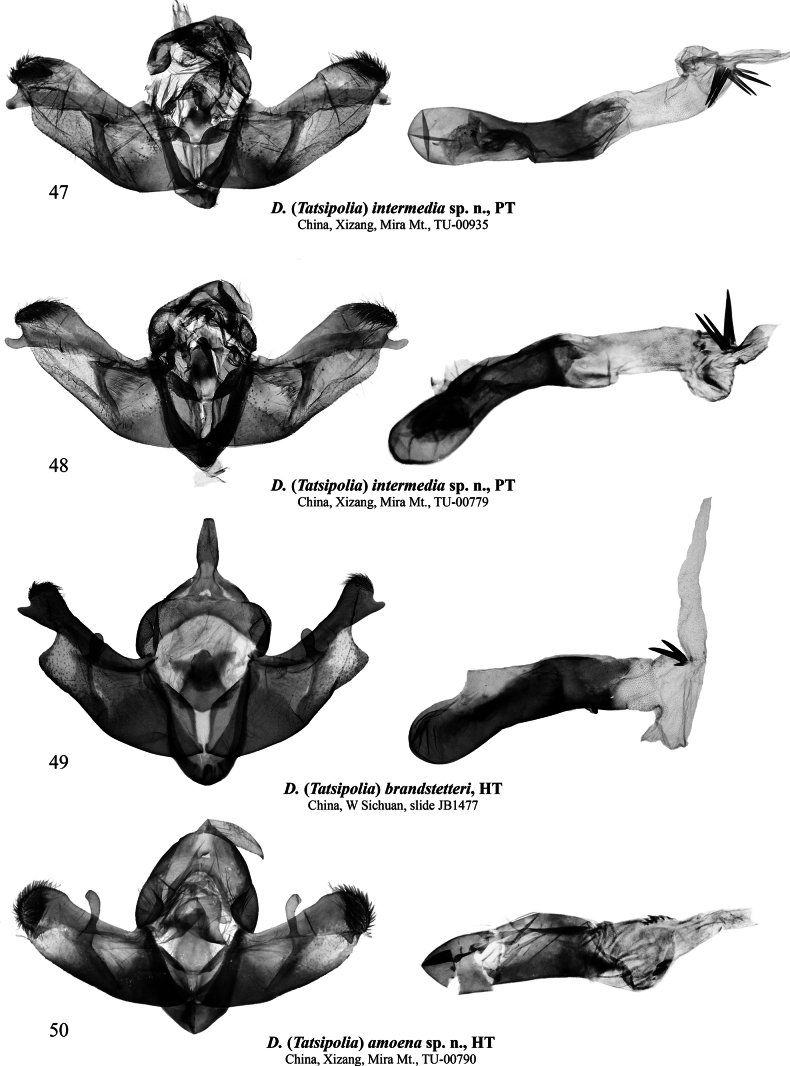
Male genitalia of Dasypolia (Tatsipolia) spp. Depositories of the specimens dissected: **47, 48, 50.** In TU; **49.** In ZSM.

**Figures 51–54.**
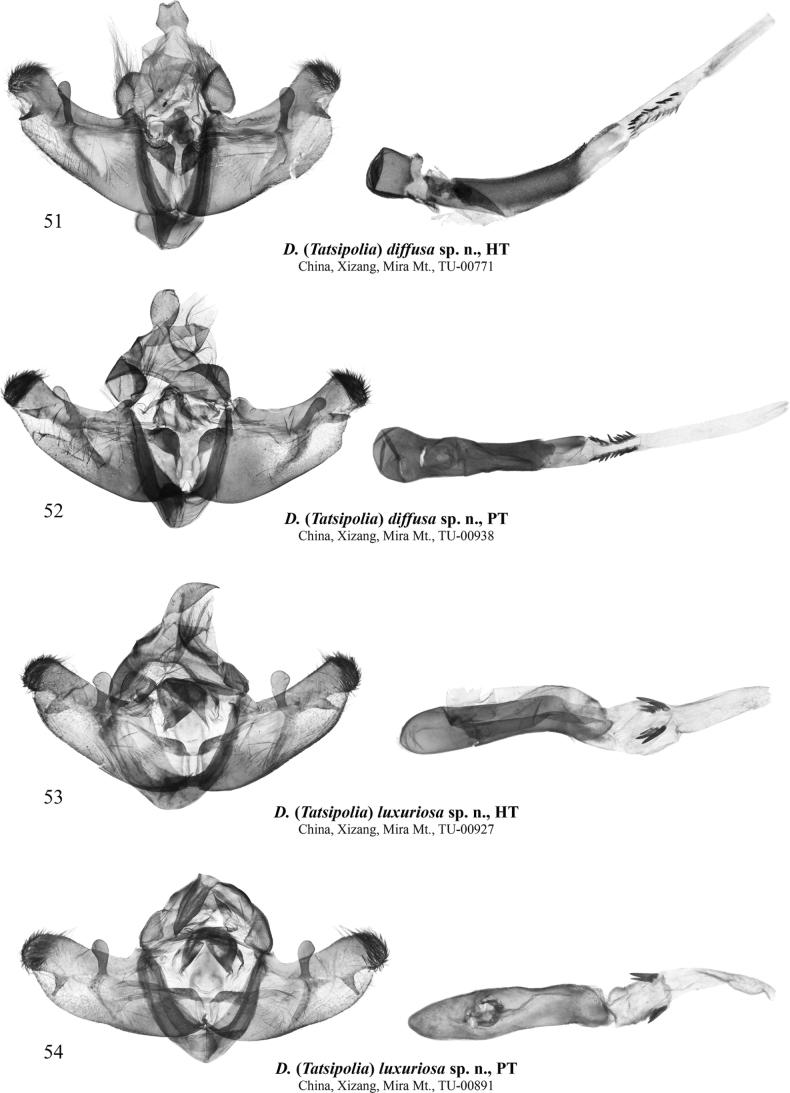
Male genitalia of Dasypolia (Tatsipolia) spp. The specimens dissected are deposited in TU.

**Figures 55–58.**
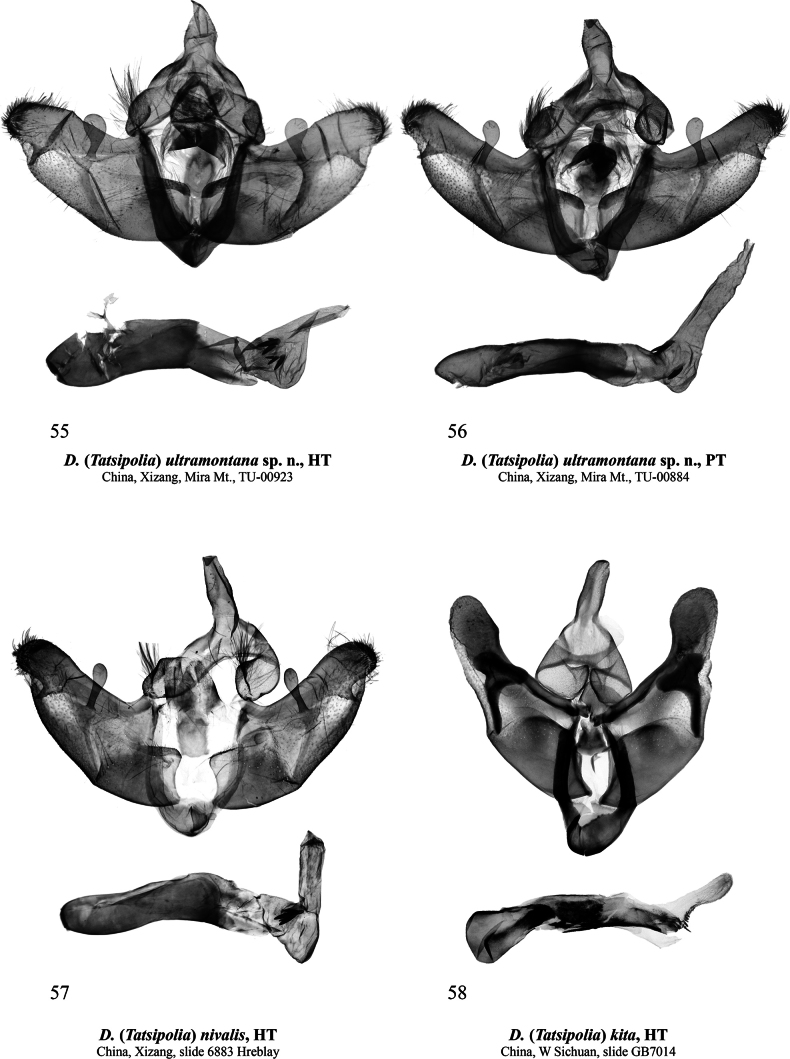
Male genitalia of Dasypolia (Tatsipolia) spp. Depositories of the specimens dissected: **55, 56.** In TU; **57.** In MH/HNHM (photo by B. Tóth); **58.** In ZSM.

**Figures 59–66.**
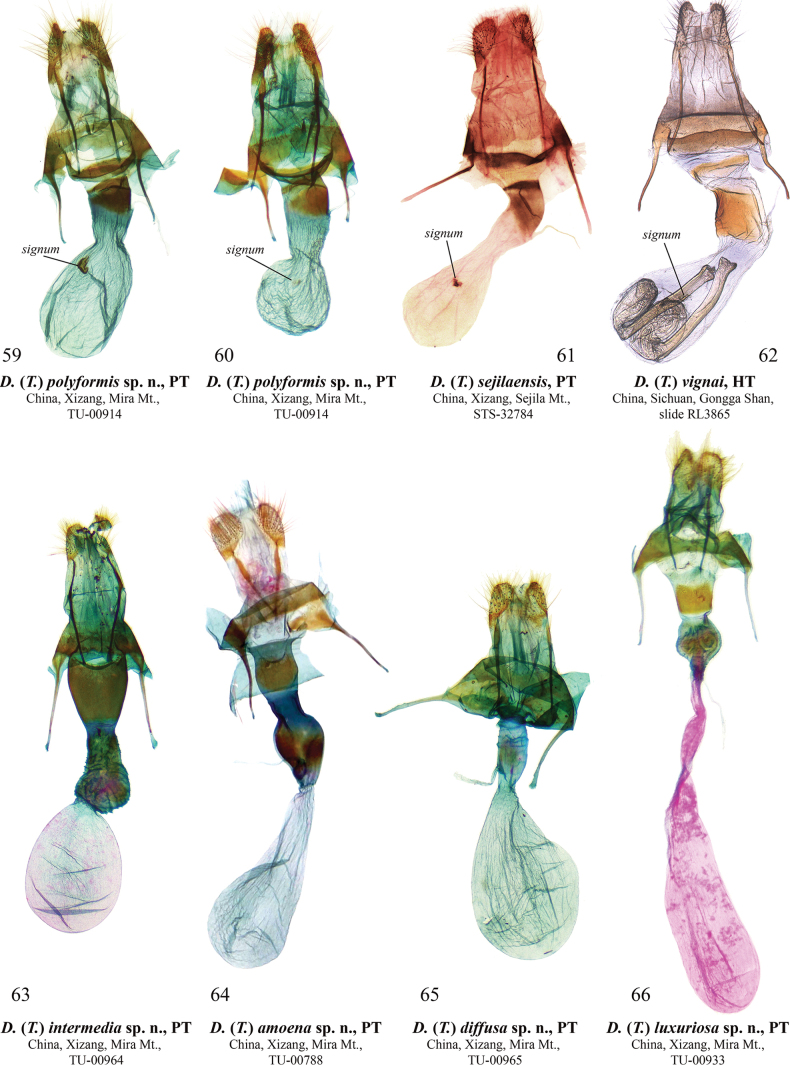
Female genitalia of Dasypolia (Tatsipolia) spp. Depositories of the specimens dissected: **59, 60, 63–66.** In TU; **61.** In TAAHU; **62.** In HNHM (photo by B. Tóth).

###### Note.

In the original description, Hreblay and Ronkay (1995) listed a D. (Tatsipolia) female specimen as paratype of *D.nivalis* and illustrated its genitalia. However, as it originates from a non-type locality, its conspecificity to the holotype is doubtful.

###### Diagnosis.

The forewing length is 13.0 mm in the male holotype. The species is morphologically similar to *D.ultramontana* sp. nov. and a detailed comparison is provided above in the diagnosis of the latter species.

###### Distribution.

The species is currently known only from its type locality in Xizang, China.

#### ﻿The D. (T.) kita species group

**Diagnosis.** The male genitalia of the species group are characterised by the combination of the basally dilated and heavily sclerotised harpe (whereas it is slender and clavate in the *D.nivalis* and reduced in the *D.vignai* species groups) and the reduction of the distal ampulla, which is well-developed in two other species groups of D. (Tatsipolia). The female genitalia are unknown.

##### ﻿ Dasypolia (Tatsipolia) kita

Taxon classificationAnimaliaLepidopteraNoctuidae

Benedek, Behounek, Floriani & Saldaitis, 2011

8D710FBB-22F7-5F84-A601-042BDBB51557

Figs 38 , 58

Dasypolia (Kitapolia) kita Benedek et al., 2011: 110, fig. 4 (male genitalia), pl. 15: fig. 7 (adult) (type locality: China, Sichuan, Kangding, near Zheduo Pass, 3700–4200 m”).

###### Type material examined.

***Holotype*** (Figs 38, 58): ♂, “China, W.Sichuan | Kangding, near | Zheduo Pass | 3700–4200 m., | 14.10.2009. | Floriani&Saldaitis leg.” / red label “Prp.Nr.: 7014 m | HOLOTYPUS Dasypolia | kita BENEDEK, BEHOUNEK | FLORIANI & SALDAITIS, | 2011 | China, Setchuan, Kangdung, | Zheduo Pass Umg., 3700–4200 m | Floriani, Saldaitis leg. 14.10.2009 | Prp. Behounek, 2011” / “coll. G. BEHOUNEK | Grafing bei München” / “BC ZSM Lep 35678” (ZSM).

###### Diagnosis.

The forewing length is 11.0 mm in the male holotype. *Dasypoliakita* is easily distinguishable from similar species by the bronze-brown thorax and forewing ground colour and the strongly bipectinate male antenna, which is similar only to *D.brandstetteri*. The key genital differences from other species in *Tatsipolia* are discussed above in the diagnosis of the *D.kita* species group. The female is unknown.

###### Distribution.

The species is currently known only from its type locality in western Sichuan Province, China.

## Supplementary Material

XML Treatment for Subgenus Tatsipolia

XML Treatment for Dasypolia (Tatsipolia) polyformis

XML Treatment for Dasypolia (Tatsipolia) cerritula

XML Treatment for Dasypolia (Tatsipolia) sejilaensis

XML Treatment for Dasypolia (Tatsipolia) vignai

XML Treatment for Dasypolia (Tatsipolia) ruficilia

XML Treatment for Dasypolia (Tatsipolia) intermedia

XML Treatment for Dasypolia (Tatsipolia) brandstetteri

XML Treatment for Dasypolia (Tatsipolia) amoena

XML Treatment for Dasypolia (Tatsipolia) diffusa

XML Treatment for Dasypolia (Tatsipolia) luxuriosa

XML Treatment for Dasypolia (Tatsipolia) ultramontana

XML Treatment for Dasypolia (Tatsipolia) nivalis

XML Treatment for Dasypolia (Tatsipolia) kita

## ﻿Additional information

### Use of AI

No use of AI was reported.

### Author ORCIDs

Enyong Chen https://orcid.org/0000-0001-7503-844X

Zhaohui Pan https://orcid.org/0000-0002-4767-7962

Anton V. Volynkin https://orcid.org/0000-0001-9447-4925

Aidas Saldaitis https://orcid.org/0000-0003-0999-3996

Balázs Benedek https://orcid.org/0000-0001-9533-1176

Yonghong Zhou https://orcid.org/0000-0002-2663-1885

### Data availability

All of the data that support the findings of this study are available in the main text.
